# Computational Approaches: An Underutilized Tool in the Quest to Elucidate Radical SAM Dynamics

**DOI:** 10.3390/molecules26092590

**Published:** 2021-04-29

**Authors:** Tamra C. Blue, Katherine M. Davis

**Affiliations:** Department of Chemistry, Emory University, 1515 Dickey Dr, Atlanta, GA 30322, USA; tcblue@emory.edu

**Keywords:** radical SAM enzymes, dynamics, computational, DFT, MD, docking, QM/MM

## Abstract

Enzymes are biological catalysts whose dynamics enable their reactivity. Visualizing conformational changes, in particular, is technically challenging, and little is known about these crucial atomic motions. This is especially problematic for understanding the functional diversity associated with the radical *S*-adenosyl-L-methionine (SAM) superfamily whose members share a common radical mechanism but ultimately catalyze a broad range of challenging reactions. Computational chemistry approaches provide a readily accessible alternative to exploring the time-resolved behavior of these enzymes that is not limited by experimental logistics. Here, we review the application of molecular docking, molecular dynamics, and density functional theory, as well as hybrid quantum mechanics/molecular mechanics methods to the study of these enzymes, with a focus on understanding the mechanistic dynamics associated with turnover.

## 1. Introduction

Carefully choreographed structural dynamics underlie enzymatic activity. However, these motions are often small in magnitude, notoriously difficult to visualize, and remain largely unexplored. Short reaction timescales and/or a lack of intermediate state accumulation complicate data collection. Furthermore, structural biology methods typically rely on cryogenic temperatures that limit study to stable states or those with lifetimes long enough for freeze trapping. Recent advancements in time-resolved crystallography show great promise for monitoring atomic changes as catalysis occurs, but there are limitations [1,2]. Many membrane proteins, as well as large or flexible molecules, for example, are difficult to crystallize [3]. Likewise, in order to study dynamical properties, the macromolecule must be biologically active in the crystalline state, the lattice must support turnover, and near simultaneous reaction initiation of all constituent molecules must be achieved. While the conformational dynamics of enzymes that rely on radical-based chemistry are of particular interest due to their ability to catalyze challenging transformations during the biosynthesis of many natural products and cofactors, these systems are often reactive to molecular oxygen, which similarly complicates handling.

**Figure 1:**
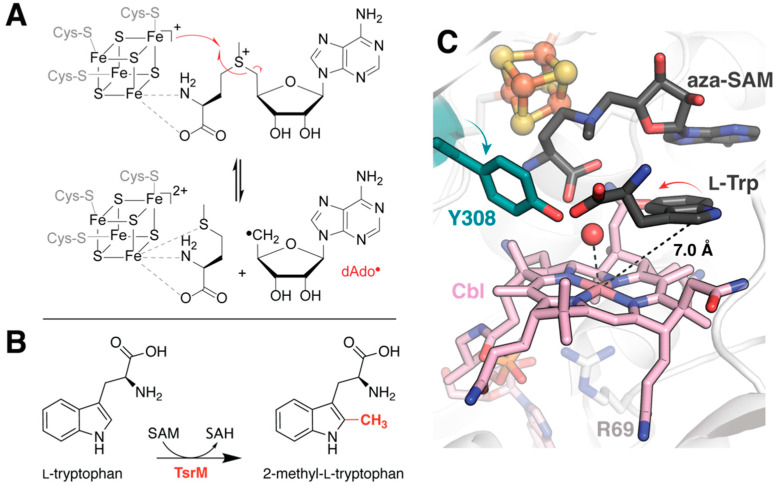
Substrate binding in TsrM. (**A**) Shared reaction of rSAM enzymes. (**B**) Reaction catalyzed by TsrM, which while annotated as a rSAM enzyme, does not appear to generate dAdo**•**. (**C**) The active site structure of TsrM (PDB accession code: 6WTF) depicts the target carbon in an unproductive position. Docking simulations suggest an alternate flipped binding mode for Trp in the presence of MeCbl. This proposal helps to rationalize observed reorientation of a nearby Tyr.

This is especially true for the >300,000 members of the radical *S*-adenosyl-L-methionine (rSAM) superfamily [4]. These enzymes catalyze a diverse array of reactions that include C–C bond formation, decarboxylation, sulfur insertion, and methylation [5]. They are bioinformatically classified via the presence of a canonical CX3CX2C motif, the thiols of which bind a catalytically-active [4Fe–4S] cluster. Homolytic cleavage of SAM bound bidentate to the unique iron of this cluster yields methionine and a 5′-deoxyadenosyl radical (dAdo**•**; Figure 1A) [5,6]. Most often rSAM enzymes employ this potent radical to abstract a substrate hydrogen, thereby enabling the enzymes’ impressive scope of reactivity. Although they comprise one of the largest known enzyme superfamilies, characterization of rSAM enzymes has been hindered by their recalcitrant nature. Computational chemistry methods provide a readily accessible alternative to studying the mechanisms and atomic motions that facilitate rSAM catalysis while overcoming the technical limitations associated with time-resolved structure determination of these enzymes.

Computational approaches have played an integral role in defining the rSAM superfamily, since its classification in 2001, when bioinformatics was used to survey and categorize the original ~600 members [7]. Computational chemistry methods that leverage our understanding of classical and quantum mechanics to analyze the fundamental properties of atoms, molecules, and chemical reactions, however, have been sorely underutilized in the study of these fascinating enzymes. Here, we summarize work that has been performed to apply both ab initio and semi-empirical calculations to the study of rSAM enzyme dynamics with a focus on docking (D), molecular dynamics (MD), density functional theory (DFT) and hybrid quantum mechanics/molecular mechanics (QM/MM) methods (Table 1). Areas of untapped potential are highlighted along with potential pitfalls.

## 2. Molecular Docking

Some of the most critical conformational changes that an enzyme undergoes are those associated with substrate binding. Accordingly, visualization of reactant complexes can provide unprecedented insight into the structural basis for reactivity. Binding of a peptidyl analog for the protein substrate pyruvate formate-lyase (PFL) by the radical SAM activating enzyme (PFL-AE) is accompanied by rearrangement of an active site beta strand, which partially loses secondary structure to provide contacts with the peptide [8]. Complexation with the PFL fragment further induces changes to side chain conformations that stabilize the cosubstrate SAM, a correlation that may help to prevent unproductive cleavage. More generally, loop motions that occlude solvent access to the active site appear to be relatively widespread amongst members of the superfamily [9,10]. Despite a shared mechanism, the vast substrate diversity of rSAM enzymes precludes a common substrate-binding motif, and conformational changes associated with ligand binding are often subtle. The structural dynamics that facilitate catalytic initiation must therefore be analyzed on a case-by-case basis. Molecular docking provides a broadly accessibly tool for evaluating this behavior that is particularly advantageous when substrate identity is unknown, the reactant complex is unstable, or co-crystallization is challenging.

Docking methods iteratively explore ligand conformations and orientations to predict the most likely complex formed with a given macromolecular target. In their simplest form, these approaches rely on the principle of shape complementarity. Resultant models are then assessed using sophisticated scoring systems which may consider physicochemical properties such as solvent effects, electrostatic interactions, and hydrophobic contacts, among other things, to provide an estimate of binding affinity [11]. Such simulations applied to rSAM enzymes have refined crystallographically-determined substrate binding modes, suggested potential dynamics associated with protein–protein complex formation, identified mechanistically relevant active site residues, and been instrumental in the evaluation of proposed substrate candidates. Relevant examples are described below.

### 2.1. Correcting for the Wrong Form of Cobalamin in TsrM

Incorporation of physiologically relevant cofactors is crucial for the accurate interpretation of structural models. However, this can be difficult to achieve, especially in systems whose cofactors require assembly, are reactive to molecular oxygen, or sensitive to light. Cobalamin-dependent rSAM enzymes unfortunately share all of these traits. Co-expression of cellular machinery for Fe/S-cluster assembly along with careful handling under anoxic conditions can secure the canonical [4Fe-4S] cluster. The incorporation and instability of methylcobalamin (MeCbl), on the other hand, has proven more problematic, and recently emerged as the primary impediment to structural characterization of a true reactant complex for tryptophan 2C methyltransferase (TsrM), annotated as a rSAM enzyme [12]. TsrM catalyzes the methylation of L-tryptophan (Trp) at the C2 position during the biosynthesis of the macrocyclic peptide antibiotic thiostrepton A (Figure 1B) [13]. Experimental structure elucidation of the TsrM•Trp complex depicts an awkwardly oriented substrate molecule, the C2 position of which is too distant (7 Å) for methyl transfer from the cobalt ion (Figure 1C). To account for this inconsistency, the authors suggest that the observed binding mode is likely due to degradation of the MeCbl cofactor to aquocobalamin during crystallization and/or data collection [12]. They utilized the Glide docking program from the Schrödinger software suite to test this hypothesis following manual replacement of the observed cofactor by MeCbl (Table 1) [14]. While the top-scoring pose recapitulates the binding conformation observed in the crystal structure, it is followed closely by a flipped conformation in which the C2 position of Trp lies approximately 3.1 Å from the methyl group of the cobalamin cofactor. In addition to providing a more catalytically relevant model for substate binding, this simulation helps to justify the rearrangement of an active site tyrosine (Y308) upon substrate binding (Figure 1C). The observed conformational change places the Tyr hydroxyl group proximal to the Trp C2. As no other suitable amino acids are nearby, it seems likely that Y308 serves as the active site base in the reaction. However, this must be verified experimentally.

### 2.2. Predicting a Structural Framework for PFL Activation

**Figure 2:**
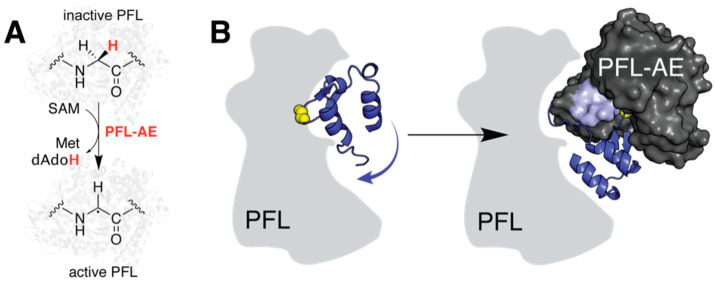
Large-scale structural rearrangements of PFL and its activating enzyme. (**A**) Reaction catalyzed by PFL-AE. (**B**) Docking studies suggest hinged motion of the radical domain in PFL (blue cartoon, PDB accession code: 2PFL) together with loop motions in PFL-AE (PDB accession code: 3CB8, mobile loop region highlighted on surface) facilitate substrate positioning. The outline of PFL is predominantly shown as a solid grey shape to help distinguish the radical domain. The catalytic glycine residue is rendered in yellow spheres for emphasis. Note the complex depicted here is not the original docking model and was generated manually for visualization of described computational results.

Although the most common applications of molecular docking involve small-molecule ligands such as Tyr, it is possible to employ docking approaches for determining protein–protein interactions [15]. Such macromolecular complexes are notoriously difficult to crystallize, and no rSAM enzyme has been structurally characterized bound to its protein substrate to date. Instead, truncated peptide mimics have been employed to investigate the structural basis for reactivity in both the anaerobic sulfatase-maturating enzyme (anSME) from *Clostridium perfringens* and PFL-AE, introduced above [16]. The latter abstracts a H atom from PFL to generate a stable glycyl radical which is used in the conversion of pyruvate and coenzyme A to formate and acetyl-CoA (Figure 2A) [17]. Vey et al. expanded upon their crystallographically obtained model using ZDOCK to predict binding to a 48-residue fragment of PFL that contains the catalytic glycine residue and is proposed to contact the activating enzyme (Table 1) [8,18]. Their results not only account for two patches of sequence conservation on the surface of PFL-AE, but further led to the proposal of concerted conformational changes in the two proteins. Namely, they hypothesize that loop motion in PFL-AE occurs in tandem with hinged motion of the radical-containing domain in PFL to facilitate binding and protect the radical post formation (Figure 2B). It should be noted that docking simulations do not perfectly duplicate the interactions observed for the peptide mimic. It is unclear whether these discrepancies are expected based on differences in *K*_M_ or whether this is a function of rigid body modeling. Computational advancements over the past decade have made incorporating ligand flexibility more accessible and subsequent studies, such as those investigating the rSAM enzyme SuiB, have benefited from this consideration [19].

### 2.3. Elucidating the Transition to Ordered Product in SuiB

**Figure 3:**
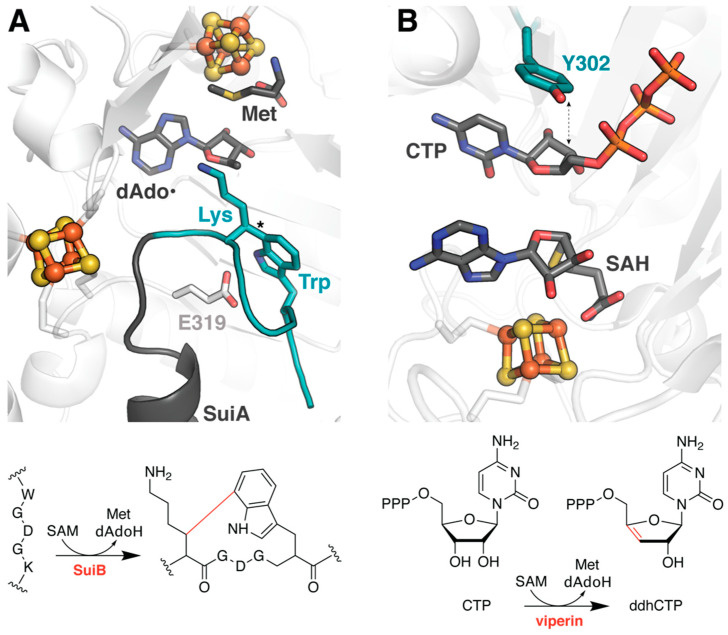
Docking studies help to identify catalytic residues in both SuiB and viperin. (**A**) SuiB catalyzes a C–C crosslink, marked with an asterisk in the structure, to cyclize the core region of a ribosomally synthesized peptide, SuiA. Docking methods were used to simulate the portion of the peptide that was disordered in the crystal structure (PDB accession code: 5V1Q), shown in teal. The putative active site base, E319, is drawn in stick representation. (**B**) Together, docking and MD studies have helped to elucidate the function of viperin (PDB accession code: 6Q2P) and support a PCET role for a conserved active site Tyr.

SuiB catalyzes the formation of an unusual C–C crosslink between lysine and tryptophan during the biosynthesis of a macrocyclic peptide natural product (Figure 3A) [20]. Unfortunately, structural characterization of the enzyme in complex with its ribosomally synthesized peptide substrate, SuiA, was insufficient to visualize the core region of the peptide containing the sites of posttranslational modification, perhaps due to unregulated turnover [19]. The authors therefore turned to molecular docking approaches carried out using Rosetta to extrapolate possible binding modes for the core sequence within the active site (Table 1) [21]. Unlike with TsrM and PFL-AE, these simulations employed a variety of constraints that not only enforced linkage to the crystallographically observed portion of SuiA, but further restricted the distance between the location of the dAdo**•** radical and the lysine Cβ, from which the H atom is abstracted. Furthermore, the FastRelax algorithm sampled backbone and side chain conformations of both the substrate and nearby active site residues to account for the inherent flexibility of the molecules [22]. While no consensus was reached for the uncyclized peptide, four possible low energy conformations were identified for the product, suggesting that the unstructured substrate core becomes ordered upon installation of the crosslink. The associated models highlight the rearrangement of side chains in the active site to accommodate product. In particular, E319 moves such that its gamma carboxyl group could, in theory, serve as the active site base (Figure 3A). The hypothesis that this titratable group is crucial for turnover was later confirmed biochemically [23].

### 2.4. Assessing the Substrate Scope of Viperin and QueE

In a more traditional application analogous to its use in the field of drug discovery, molecular docking has also been used to evaluate proposed substrates of the rSAM enzyme viperin (virus inhibitory protein, endoplasmic reticulum-associated, interferon-inducible; Table 1). Viperin orthologs are ubiquitous across all domains of life, including in humans, and have been demonstrated to inhibit viral replication [24,25]. The molecular mechanism underlying this antiviral activity, however, was only recently determined [26]. From numerous promising candidates, including those supported by computational docking [27,28], the mammalian enzyme was ultimately shown to catalyze the conversion of cytidine triphosphate (CTP) to the RNA-chain terminator 3′-deoxy-3′,4′-didehydro-CTP (ddhCTP), as shown in Figure 3B [26]. Subsequent docking models helped to investigate the role of an active site tyrosine in a key proton-coupled electron transfer (PCET) step of this reaction and provided evidence to substantiate the divergent substrate promiscuity of fungal viperin from *Thielavia terrestris* (TtRSAD2) [29]. Molecular dynamics simulations extended these studies to elucidate associated conformational dynamics and are described below.

**Figure 4:**
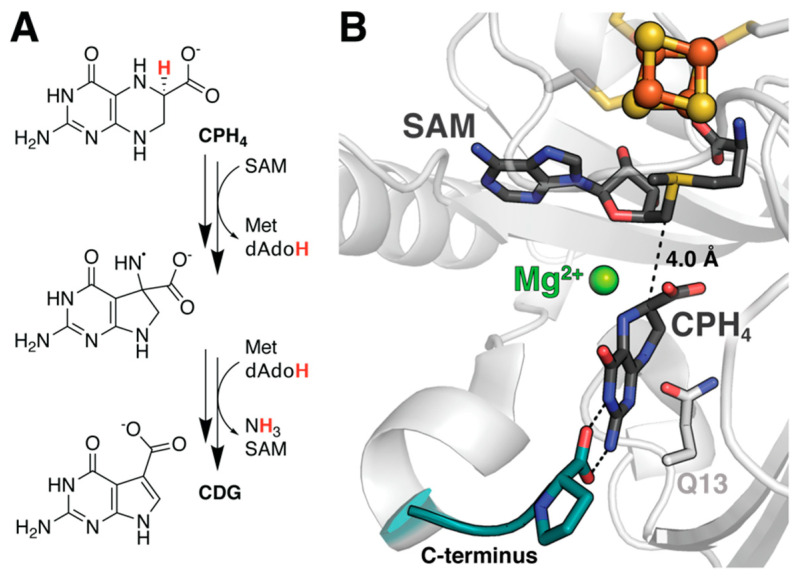
Subtle structural features of the QueE reactant complex are crucial for enzyme reactivity. (**A**) Reaction scheme for QueE. (**B**) A series of docking, MD, DFT, and QM/MM studies have demonstrated the importance of divalent ion identity and substrate deformation to both stabilize the reactant complex and facilitate catalysis. Decreased flexibility in the C-terminal tail was also observed following reaction initiation in QueE (PDB accession codes: 4NJI).

In fact, molecular docking is often used as a preparative step for downstream MD or QM/MM analysis, and a similar evaluation of substrate scope was performed for 7-carboxy-7-deazaguanine synthase (QueE) from *B. multivorans* with an eye toward engineering alternative reactions [30]. QueE catalyzes the Mg^2+^-dependent rearrangement of 6-carboxytetrahydropterin (CPH_4_) to 7-carboxy-7-deazaguanine (CDG), the basic building block for more than 30 natural products in addition to the tRNA base queuosin (Figure 4A) [31]. Docking approaches performed in tandem with DFT calculations of radical stabilization energies demonstrated that either less stabilization or the ability to persist in a non-preferred conformation is required for ligands to serve as alternative substrates of this enzyme. As with viperin, these studies were enhanced by MD simulations detailed below.

## 3. Molecular Dynamics

Although molecular docking can certainly inform our understanding of enzyme dynamics, it is limited to providing static snapshots of ligand-bound complexes. This is problematic, as enzymes are constantly in motion, their atomic rearrangements a concerted dance precisely tailored to propel the reaction forward. For the computational prediction and analysis of these time-dependent conformational changes, we turn to molecular dynamics. MD utilizes classical mechanics approaches, based on Newton’s laws, to simulate atomic motion. The forces acting on each atom are most commonly calculated using molecular mechanics (MM) force fields that approximate atoms as charged spheres, and the covalent bonds between them as springs [32]. These assumptions reduce the computational cost associated with ab initio calculations on macromolecular systems. Resultant MD trajectories can then be used to assess the magnitude of structural changes (root-mean-square deviation [RMSD]), the propensity for a given residue or region to move (root-mean-square fluctuation [RMSF]), and the evolution of hydrogen bonding networks, among other things [33]. Despite its utility, few groups have applied MD for the study of rSAM enzymes, and those that have primarily focused on better understanding the requirements for substrate binding. In fact, one of the first MD studies on a member of the rSAM superfamily essentially utilized the disproportionately powerful technique for the purposes of docking possible substrate candidates to viperin.

### 3.1. The Flexible Binding Pocket of Fungal Viperin

Although all viperin orthologs tested thus far appear to catalyze the conversion of CTP to ddhCTP, substantial experimental evidence points to the existence of additional alternate reactivities in both fungal- and archaeal-derived enzymes [29,34]. Prior to identification of CTP as the primary substrate, Chakravarti et al. observed apparent modification of isopentenyl pyrophosphate (IPP; Table 1) [34]. To evaluate whether there was a structural basis for IPP binding to the enzymes, they manually placed the ligand in the active site of a crystallographically characterized mouse viperin model and performed successive rounds of MD equilibration. The resultant complex identified numerous side chain rearrangements that could enable interactions with the terminal phosphate of IPP, consistent with the authors’ biochemical analysis of substrate scope. Perhaps more compelling in hindsight, however, is the simulated orientation of IPP, which places the hypothesized substrate radical on C3 near the putative catalytic tyrosine, identified later via docking models with CTP (Figure 3B) [29]. Such proximity not only supports the proposed mechanism for IPP reactivity, but further highlights the significance of the active site tyrosyl.

MD simulations following docking of uridine triphosphate (UTP), another putative fungal viperin substrate that has been identified biochemically, to TtRSAD2 reportedly indicate significant flexibility in the loops responsible for binding the ribose moiety (Table 1) [29]. This conformational elasticity is in agreement with electron paramagnetic resonance data demonstrating that interaction with the sugar induces detectable structural changes likely important for facilitating catalysis. Furthermore, the position of the uracil moiety fluctuates within the binding pocket consistent with an insensitivity to the nucleobase identity and perhaps the origin of the enzyme’s intriguing promiscuity.

### 3.2. Substrate Stabilization in QueE

MD simulations have likewise been used to assess the stability of alternative substrates within the active site of QueE post-docking (Table 1). This analysis was critical due to the electrostatic effects of a catalytic Mg^2+^ ion that stabilize a bent and energetically unfavorable configuration of the substrate vital for reactivity (Figure 4B) [35,36]. Given this requirement, docking models with appropriate positioning and radical stabilization energies (RSEs) are still insufficient to validate a given molecule as a substrate. Suess et al. demonstrated that MD trajectories of seemingly suitable protein–ligand complexes in the absence of Mg^2+^ showed rapid dissociation, reiterating the importance of the ion for complex stability [30]. Retention times of the natural substrate CPH_4_ were also reduced upon replacement of Mg^2+^ by other ions, including Ca^2+^ and Na^+^, likely because they fail to maintain the same hydrogen bonding network. It should be noted that the incorporation of MD to perform such equilibrium studies following any docking procedure is good practice. Finally, MD was applied to gain insight into the behavior of a key catalytic intermediate. The resultant data indicate increased rigidity of the C-terminal tail along with a tighter hydrogen bond to the substrate upon evolution from the reactant complex (Figure 4B). Such subtle changes in atomic position and reduced capacity for motion, leading to reduced loop flexibility, are difficult to visualize experimentally, but can provide crucial insights into enzymatic turnover.

## 4. Density Functional Theory

The computational approaches described thus far have examined conformational dynamics that, while crucial, cannot solely account for enzymatic reactivity. Modulation of active site electronic structure must occur in tandem to effect turnover. Molecular mechanics approximations, however, are unable to elucidate these changes. Furthermore, methods dependent on such approximations are ill equipped to accurately evaluate proposed intermediate states based on the total energy. Ab initio or pseudo ab initio quantum mechanical (QM) methods can overcome these limitations and enable the properties of multi-electron systems to be determined. DFT, for example, allows one to calculate electronic properties by correlating the ground state energy with the spatial density of electrons [37]. This powerful strategy reduces the computational cost compared to wavefunction-based methods. DFT can provide insight into the thermodynamic and kinetic properties of a given system, including reaction energies, charge/spin distributions, and vibrational frequencies, in addition to molecular geometries [37,38]. Perhaps unsurprisingly, it is the most common computational method used to analyze the behavior of rSAM enzymes. Herein, we will focus on a subset of those studies that address the dynamics associated with catalysis.

### 4.1. Probing the Basis for SAM Cleavage in PFL-AE

Early electron nuclear double resonance (ENDOR) studies of PFL-AE observed orbital overlap between the SAM sulfonium and the canonical Fe/S cluster, thereby providing the first evidence to suggest that SAM binds directly to the unique Fe in radical SAM enzymes [39,40]. To further explore the nature of this interaction and the role of the cluster in SAM cleavage, Dey et al. used DFT calculations to assess the composition of the molecular orbitals and evaluate potential energy surfaces (Table 1) [41]. Resultant data were used to rationalize an increase in the sulfur X-ray absorption pre-edge intensity upon SAM binding and validate hypotheses that this feature arises due to backbonding between the cluster and the S-C5′ σ * orbital of SAM [42]. Calculations suggest that both reduction of the cluster and precise positioning of SAM enhance this effect to facilitate homolytic bond cleavage. Intriguingly, recent experimental studies indicate that thermally induced conformational changes are likely responsible for regioselectivity of the S-C5′ bond [43]. The analysis of potential energy surfaces by Dey et al. also identified active site polarity as a key component to modulating the activation energy required for reductive SAM cleavage [41]. Namely, a decrease in polarity is correlated with a lower barrier. The authors therefore propose that substrate binding may trigger generation of dAdo**•** by decreasing solvent accessibility to the active site, which would lower the dielectric constant in the medium. Such a gating mechanism may also help to mitigate unproductive cleavage in the absence of substrate.

### 4.2. Analyzing the Effect of Ion Complexation to QueE

While DFT was used to assess the energetics associated with the characteristic radical SAM cleavage reaction in PFL-AE, a similar approach can be used to elucidate the basis for primary substrate modifications. This is of particular interest for enzymes that use radicals to perform selective modifications, as radical reactivity is difficult to control. DFT, towards this end, has been employed in the study of QueE from *B. multivorans* (Table 1) [36]. Calculated radical stabilization and rearrangement energies associated with the modification of CPH_4_ support the QM/MM-derived hypothesis that electrostatic interactions with Mg^2+^ induce a strained substrate conformation important for catalysis (Figure 4B) [35]. Obtained results further indicate that the metal ion not only promotes deviation from substrate planarity, but must also maintain this energetically unfavorable conformation upon generation of the radical via H-atom abstraction in order to lower the barrier for rearrangement. This effect is diminished upon complexation with other divalent ions, in agreement with slower kinetics and reduced activity observed experimentally. Although distortion of the substrate destabilizes the radical, thereby increasing the probability of undesirable off-pathway reactions, the presence of Mg^2+^ tunes the energy landscape to preclude unproductive ring opening.

### 4.3. Determining Transition State Dynamics in MoaA

**Figure 5:**
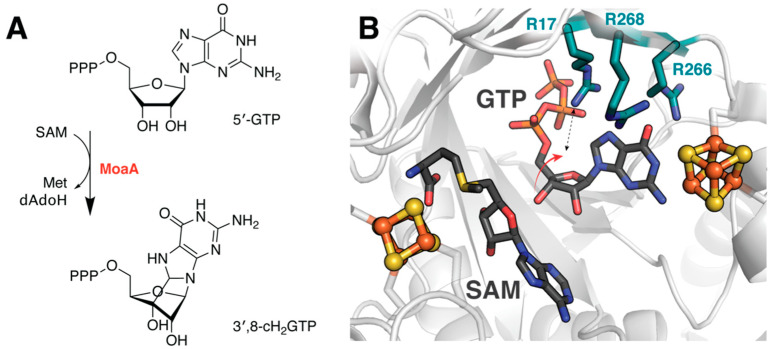
Active site residues facilitate MoaA catalysis. (**A**) Reaction catalyzed by MoaA. (**B**) DFT studies indicate that arginine residues lining the MoaA substrate binding pocket play a role in modulating reaction energetics. These calculations further suggests that reorientation of the ribose moiety enables a key interaction with Arg17 to stabilize the transition state. Structure shown from PDB accession code 2FB3, excluding SAM, which was aligned from 1TV8.

Enforcement of a nominally unfavorable substrate conformation to overcome otherwise high activation barriers was also discovered in the rSAM enzyme MoaA, which cyclizes guanosine triphosphate (GTP) to generate 3′,8-cyclo-7,8-dihydro-GTP (3′,8-cH_2_GTP) during biosynthesis of the essential molybdenum cofactor, Moco (Figure 5A) [44]. DFT-based analyses reveal that constraining both the nucleobase and triphosphate moiety, consistent with observed hydrogen bonding interactions between active site constituents, facilitates deformation of the substrate (Table 1) [45]. The resultant molecular arrangement decreases the activation energy of cyclization. However, it does not fully account for the experimentally derived kinetics. Instead, the presence of three arginine residues that line the substrate binding pocket lower the barrier to within range of estimated activation energies (Figure 5B). Interactions with these residues, however, were only observed in geometry optimizations of the transition state complex which depict the 3′-OH of the ribose pivoting to form a hydrogen bond with Arg17. It seems likely that this dynamic behavior is crucial to allow the reactant to pass through the transition state. Associated DFT calculations that demonstrate comparable thermodynamic free energies for the reactant and product complexes were also proposed to support involvement of the C-terminal auxiliary cluster in dynamic redox processes associated with MoaA catalysis.

### 4.4. Beyond Dynamics

Although the examples presented above highlight the importance of structural changes to effect catalysis, DFT has also been applied to evaluate proposed mechanistic pathways [46,47,48], assist in the analysis of spectroscopic data [49], and compare the thermodynamic reaction profiles of enzymes within the superfamily [50].

## 5. Hybrid Methods: Quantum Mechanics/Molecular Mechanics

Quantum mechanical approaches such as DFT are incredibly powerful, but they are impractical on a large scale due to their high computational cost. However, consideration of only a subset of atoms in the active site can be equally problematic as this neglects the influence of the broader protein environment on reaction energetics. Hybrid QM/MM methods attempt to overcome these obstacles by utilizing more accurate ab initio calculations to simulate important electronic structure features, while handling the rest of the molecule classically. Approximating interactions between most atoms with a simple potential energy function dramatically increases computational tractability. Practically, this means assigning different regions of the protein to QM or MM treatment, as well as evaluating the electrostatic effect of the MM region on the QM components [51,52]. It should be noted that a short MD simulation is typically performed to equilibrate molecules prior to initiating QM/MM calculations, and QM regions are most often restricted to active site features that directly impact reactivity. As a frame of reference, studies of QueE isolated only the CPH_4_ substrate, dAdo**•**, and six active site residues, along with the key Mg^2+^ ion and a series of associated waters for quantum mechanical consideration [35]. Results of these and select other QM/MM calculations aimed at elucidating the dynamic changes that facilitate radical SAM enzyme catalysis are described below.

### 5.1. Coordinated Changes to the Active Site in QueE

We have already discussed the results of QM/MM methods applied to QueE in passing to contextualize studies employing other computational techniques. In particular, this hybrid approach helped to clarify the role of Mg^2+^, as well as eliminate ring opening as a pathway to ring contraction during the production of CDG (Table 1). The discovery that the metal ion did not serve a catalytic function, but rather provided key interactions with substrate that are important for generating the reactive complex, provided the basis for subsequent studies. Zhu and Liu also observed reorientation of several active site residues in the absence of substrate/product [35]. Simulations depict the formation of an additional hydrogen bond between two glutamate residues (E15 and E116) that the authors propose forms part of a proton transfer pathway required to regenerate the active state following dissociation of the CDG product. Likewise, a nearby glutamine (Q13) side chain rotates substantially, suggesting a potential role in controlling product release (Figure 4B). It remains to be seen whether these dynamics are physiologically relevant. However, a more recent structure of the orthologous QueE from *B. subtilis*, solved in the absence of substrate, does show similar rotation of the analogous residue, perhaps supporting this hypothesis [53].

### 5.2. Proton Transfer Precedes Methyl Transfer in RlmN

**Figure 6:**
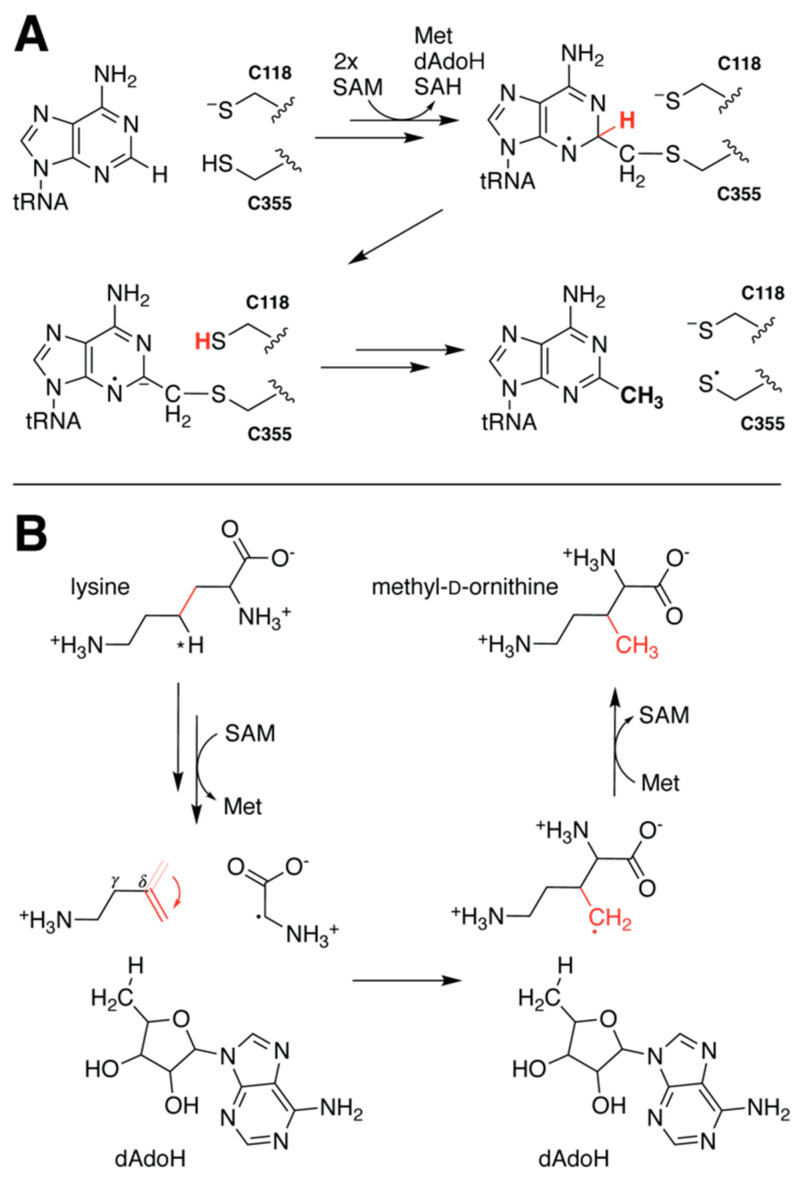
Mechanistic dynamics of RlmN and PylB elucidated by hybrid computational methods. (**A**) QM/MM calculations of RlmN indicate that deprotonation of the target carbon likely precedes cleavage of the tRNA-Cys crosslink. (**B**) Study of the PylB mechanism via hybrid methods led to the proposal that reorientation of the aminobutene intermediate (indicated by the red arrow) prior to recombination facilitates re-abstraction of the H atom from dAdoH.

Hybrid methods have also been used to determine the order of events during catalysis of the rSAM methyltransferase RlmN. This putative ‘housekeeping’ enzyme modifies key adenosine nucleosides of both transfer and ribosomal RNA and has been intensively studied in part due to its high homology with other members of the superfamily known to confer antibiotic resistance [54]. Unlike most SAM-dependent methyltransferases, RlmN utilizes SAM as both a methyl donor and a source of dAdo**•**. The methyl group is first transferred to an active site cysteine via an S_N_2 mechanism. It is then appended to the RNA substrate by means of radical addition following reductive cleavage of the second SAM molecule (Figure 6A) [55]. To address discrepancies between the starting model of earlier DFT calculations [56] and a subsequent structure of a crosslinked RlmN•tRNA complex [57], Zhao et al. applied QM/MM methods to refine the proposed mechanism (Table 1) [58]. By evaluating the energy barriers and potential energy surfaces for different reaction pathways, the authors were able to assign deprotonation of the target carbon as the rate-limiting step. They further concluded that this step precedes cleavage of the crosslinked intermediate state complex to generate a more complete picture of mechanistic dynamics (Figure 6A).

### 5.3. Rotation of Intermediate Crucial for PylB Reactivity

A similar study investigated the mechanism of methylornithine synthase (PylB), known to catalyze the rearrangement of lysine to methyl-D-ornithine during the biosynthesis of the 22nd amino acid pyrrolysine [59]. Earlier structure solution of PylB in complex with product appeared to support a fragmentation–recombination mechanism in which hydrogen atom abstraction from lysine Cγ fractures the molecule generating a glycyl radical and aminobutene that later reunite [60]. QM/MM calculations performed on a lysine docking model enabled investigation of the proposed reaction pathway energetics (Table 1) [61]. Although the study primarily focused on evaluating energy barriers for the assessment of mechanistic feasibility, the authors did observe unanticipated dynamics of the aminobutene intermediate. Rotation along the original lysine Cγ-Cδ bond, while not explicitly required for recombination, was observed to facilitate return of the proton from dAdoH. Such a reorientation dramatically reduces the distance between the newly formed Cβ radical and the C5′ of dAdoH in preparation for re-abstraction of the previously transferred hydrogen (Figure 6B). To the best of our knowledge this rotation has yet to be experimentally validated, but computational evidence for such behavior highlights once more the importance of dynamics in enzyme reactivity.

**Table 1:** Summary of computational approaches applied to rSAM enzymes discussed herein.

Enzyme	Organism	PDB Code	Computational Method/s
TsrM	*Kitasatospora cheerisanensis*	6WTE, 6WTF	D [12]
PFL-AE	*Escherichia coli*	3C8F, 3CB8	D [8] DFT [41]
SuiB	*Streptococcus suis*	5V1Q	D [19]
Viperin	*Mus musculus* *Trichoderma virens*	5VSL, 5VSM, 6B4C	D [27,28,29] MD [29,34]
QueE	*Bacillus multivorans*	4NJI, 4NJK	D, MD, DFT, QM/MM [30,35,36]
MoaA	*Staphylococcus aureus*	1TV8, 2FB3	DFT [45]
HydE	*Thermotoga maritima*	3CIW	DFT [50]
RlmN	*Escherichia coli*	5HR6	DFT, QM/MM [56,58]
PylB	*Methanosarcina barkeri*	3T7V	QM/MM [61]

## 6. Conclusions

In conclusion, the case studies presented here provide an overview of molecular docking, MD, DFT and hybrid methods applied to the study of rSAM enzymes to date. Experimental approaches are inherently unable to observe transition state species, and the short-lived nature of catalytic intermediates likewise complicates their characterization. Computational methods, by contrast, provide a unique opportunity to elucidate the atomic and electronic changes that facilitate catalysis irrespective of kinetics. Despite their application to the systems described herein, computational methods have been underutilized in the study of rSAM enzymes, due in part to complications associated with implementation. At the most basic level, the feasibility of these studies relies on the availability of reliable starting models, which are currently lacking for the majority of this superfamily. Furthermore, computational treatment of transition metal ions and organic radicals is often challenging. Of particular note are the poor performance of DFT and other QM methods applied to transition metal chemistry, as well as ambiguity in the parametrization of Fe/S clusters for docking/MD simulations.

Nonetheless, there is great potential. This is especially true for molecular dynamics approaches, which have the ability to provide insight into large-scale motions necessary for turnover, but have primarily been implemented for pseudo-docking simulations. Such methods would likely be especially useful in the study of multi-modular rSAM enzymes that contain auxiliary domains beyond the canonical triose-phosphate isomerase (TIM) or modified TIM-barrel fold characteristic of the superfamily. Indeed, most constituent members are comprised of two or more structural domains that contribute to their functional diversity, often by binding additional cofactors, or providing crucial interactions with substrate [62]. As we continue to learn more about this fascinating superfamily, the opportunities to apply computational approaches to generate and investigate experimental hypotheses will only continue to grow. Careful application of these methods together with biochemical validation position them as powerful predictors of rSAM enzyme dynamics.

## Data Availability

Not applicable.
